# Editorial

**Published:** 1838-11-07

**Authors:** 


### THE MEDICAL EXAMINER.

PHILADELPHIA, NOV. 7, 1838.

The tone and spirit of the American medical press is, in general, unexceptionable. The freest expression of opinion is shown to be compatible with decorum and courtesy of language. It is felt that personalities are unworthy the dignity of our professional literature, and if, here and there, an outbreak of ill temper and scurrility emanates from some turbulent spirit, the contempt awarded him proves the general correctness of taste and feeling.

Sectional feeling is, we are glad to say, seldom evinced by the professional periodicals of our country. The appeal to local prejudices, in a literary discussion, is paltry. It degrades the scientific to the level of the partisan political journal, and, though it may succeed in effecting particular private ends, it is visited with the disapprobation of every candid and enlightened mind. Happily, our remarks have no application to the majority of our cotemporaries. If they have some point, in a solitary instance, for the honour of the profession, we regret it. We trust that the party in question will profit by the rebuke of a journal of its own vicinity; and, for the future, abstain from "introducing this species of unjust and unmanly, or, to say the least, *unscientific* writing into a medical journal."

---

The introductory Lectures of the Medical Schools of this city, began on the 5th of November. A short course was given at both these institutions during the month of October; but the integrity of the winter course has not been interfered with—the October lectures being quite distinct, and for the most part confined to such subjects as are not taught during the winter.

A series of lectures will be given at the Philadelphia Hospital, upon Clinical Medicine and Surgery, on the Saturday of each week. The lectures on Surgery will be given by Dr. Gibson, and those on Medicine by Drs. Jackson and Gerhard. On each Saturday, the students who have purchased the tickets are conveyed to the Hospital without additional charge. On the other days of the week, especially on Wednesday, the patients will be visited in the wards; and demonstrations of the symptoms, and of the mode of treating diseases, will be given by the physicians in attendance. It is intended to render the course of lectures as systematic as possible, and to arrange them in such an order as to make it easy for the pupils to understand the mode of observation required to pass from the more simple to the more difficult diseases. The lectures are conducted in the following manner:—

The hospital is built upon such a plan, that the area of the lecture room is on the same floor with the principal story of the building; patients are carried on their beds, through the corridor which communicates with the wards, to the lecture room. The symptoms of the disease are then pointed out, and the prescription is made in presence of the class. The result of the case is always made known to the pupils, and regular notes are kept of the changes that occur during their absence. This mode of presenting the patients to a large class, is by far the best that can be adopted. The wards of a hospital cannot be visited with advantage by a class more numerous than thirty or forty students. Twenty is perhaps as large a number as can enjoy all the advantages to be obtained from a visit to each patient. But when the patients are introduced into the spacious lecture room at the Philadelphia hospital, they are not unnecessarily fatigued, and the students enjoy every advantage which they can obtain from clinical medicine, except a knowledge of those modes of investigation which require personal examination of the patients. These methods of investigation cannot, of course, be taught to a large class.

The obstacles which prevent this system from becoming more effective, depend upon the shortness of the course and the infrequent visits of the students to the hospital. For these evils there can be no remedy until students are willing and able to prolong their studies, and to direct their individual attention to a systematic course during the whole number of years which they devote to the study of medicine. All that can be done at present, is to render the system of clinical instruction as effective as is compatible with the existing opinions and practice of the students of medicine. The effort to bring about a more thorough education should not be lost sight of; nor do we intend to forget that our duty as editors requires us to insist upon the necessity of a gradual, but progressive improvement.

The greater part of the class attending the hospital, is composed of the students who enter a medical school for the first time. This arises, of course, from the necessity which more advanced students feel or believe they feel of devoting their whole time to the preparations for examination. As clinical medicine requires a certain degree of previous knowledge, and forms as it were, the complement of a medical education, numerous disadvantages result from the imperfect previous qualifications of many of the pupils. We are convinced that the really qualified pupils commit a pernicious error, in neglecting instruction of a practical kind, and in preferring to devote the best portion of their time to details which are obviously of less permanent advantage, than a knowledge of those practical departments of their profession which are to become the business of their lives. Students who are not yet fully prepared to understand all the facts which are presented to them, in a course of clinical medicine, cannot do better than to attend carefully, note down what they learn, and in their moments of leisure make themselves familiar with the deductions which these facts may afford them. When their memory is well stored with the phenomena of disease as they occur in nature, reading upon the practice becomes a most agreeable and instructive study, instead of being an arid and objectless pursuit. They have before them tangible objects with which they may connect the statements of authority, and they are prepared to reason upon what they read, instead of trusting only to a mere effort of the memory.

One word more seems necessary in explanation of these remarks, in urging upon students the necessity of attending a course of public lectures, with which one of us is connected; we should explicitly state, that no part whatever of the fee paid by students, is received by the lecturers. It is solely for the use of the institution, after deducting the expenses of the line of carriages, and also the sums paid for keeping up a large library for the use of the pupils.

We have not spoken of the clinical instruction given at the Pennsylvania hospital, the oldest institution of the kind in the United States. The buiding is not so well adapted for the purpose of instruction as the Philadelphia hospital, nor are the medical patients equally numerous. The Pennsylvania hospital is especially useful as a school for surgical operations and recent accidents. We hope, however, from time to time, to add greatly to the interest of our columns, by reports of the excellent lectures which are there given, both on medicine and surgery. Our readers are already informed of the numerous interesting surgical cases, constantly occurring in that institution; whilst the lectures of its medical attendants, which we have occasionally published, show that they are earnestly and usefully engaging in diffusing a knowledge of practical medicine and surgery.

